# Differences in Leaf Flammability, Leaf Traits and Flammability-Trait Relationships between Native and Exotic Plant Species of Dry Sclerophyll Forest

**DOI:** 10.1371/journal.pone.0079205

**Published:** 2013-11-18

**Authors:** Brad R. Murray, Lyndle K. Hardstaff, Megan L. Phillips

**Affiliations:** School of the Environment, University of Technology Sydney, New South Wales, Australia; University of Konstanz, Germany

## Abstract

The flammability of plant leaves influences the spread of fire through vegetation. Exotic plants invading native vegetation may increase the spread of bushfires if their leaves are more flammable than native leaves. We compared fresh-leaf and dry-leaf flammability (time to ignition) between 52 native and 27 exotic plant species inhabiting dry sclerophyll forest. We found that mean time to ignition was significantly faster in dry exotic leaves than in dry native leaves. There was no significant native-exotic difference in mean time to ignition for fresh leaves. The significantly higher fresh-leaf water content that was found in exotics, lost in the conversion from a fresh to dry state, suggests that leaf water provides an important buffering effect that leads to equivalent mean time to ignition in fresh exotic and native leaves. Exotic leaves were also significantly wider, longer and broader in area with significantly higher specific leaf area–but not thicker–than native leaves. We examined scaling relationships between leaf flammability and leaf size (leaf width, length, area, specific leaf area and thickness). While exotics occupied the comparatively larger and more flammable end of the leaf size-flammability spectrum in general, leaf flammability was significantly correlated with all measures of leaf size except leaf thickness in both native and exotic species such that larger leaves were faster to ignite. Our findings for increased flammability linked with larger leaf size in exotics demonstrate that exotic plant species have the potential to increase the spread of bushfires in dry sclerophyll forest.

## Introduction

Fires shape the ecology and evolution of many plant communities throughout the world [1]–[3]. There is growing concern that substantial increases in bushfires predicted under future climate-change scenarios [4], [5] will have serious ecological consequences including declines in biodiversity and modifications to ecosystem function [6]–[10]. There is also the troubling prospect that the ecological impacts of increased bushfire coverage might be compounded by increased bushfire intensity and frequency in systems where introduced exotic plant species have become established [11]–[14]. Such situations will only arise if exotic plants have intrinsic fuel properties that differ from those of native plants, properties such as increased flammability that enhance the spread of fire.

The ability of bushfire to spread through vegetation depends largely on plant flammability which varies widely among species [15], [16]. Although all plants will likely burn in extreme bushfire conditions [17], the rate of ignition of fuel (i.e. ignitability) is thought to be one of the most important factors in determining plant flammability and bushfire spread [18]. In particular, plant leaves are considered to be the most important flammable plant structure [19], [20] because leaves are frequently the first structures to ignite during bushfire [18], thus promulgating fire to other plant structures and fuel sources. However, identifying how variation among species in functionally important traits such as leaf size relates to variation in leaf flammability is still in an early stage [21].

Determining generalities in the context of plant flammability-trait relationships is difficult at present as different studies have adopted different approaches, all with their own merits. For instance, some studies have measured leaf flammability as ignitability [19]–[23], which is the time to first flaming from the time of first exposure to an ignition source, while others have used flammability measures including extinguishment of combustion of plant parts [14], [24], energy content as an indication of combustibility [16], heat flux from burning leaves [25] and large-scale fire severity scores of plant assemblages from field-based assessments of fire history [21]. Furthermore, some studies have determined flammability of individual leaves to model separated canopy leaves suspended above a surface fire [19] while others have used average leaf size of species within sampling areas [21] and either mono-specific litter beds or litter beds composed of mixtures of litter from different species [25], [26] in laboratory-based burning trials to model leaf-litter flammability dynamics.

Despite differences among studies in their approaches, tantalizing evidence is emerging that leaf size might be an important correlate of plant flammability, irrespective of the model of flammability under investigation. For instance, leaf length has been linked to flammability with longer leaves associated with higher local fire severity [21]. Larger leaves tend to create an open litter-bed structure that burns more rapidly because of better ventilation [25]. In addition, leaf water content might be an important correlate of leaf flammability, because a leaf will generally not ignite until most of its moisture is lost through evaporation [19]. Consequently, plant species with high moisture content might be less flammable as they take longer to ignite [19]–[23]. A large, comparative study across a broad taxonomic spread of native and exotic species would provide an important test of relationships between leaf size, water content and plant leaf flammability.

In this study, we determine whether leaves of exotic plant species are more flammable than those of native plant species. We compare both fresh-leaf and dry-leaf flammability between native and exotic plant species inhabiting dry sclerophyll forest in south-eastern Australia. Dry sclerophyll forests are prone to fires that can spread via leaves in the canopy and in the leaf litter [27]–[29]. We also compare several traits of plant leaves (length, width, area, thickness, specific leaf area and % water content) between native and exotic species to test whether observed native-exotic differences in leaf flammability might be underpinned by differences in leaf size and % water content. We then test whether leaf size and % water content are correlated with interspecific variation in leaf flammability. Through the use of standardized major axis regression [30] we quantitatively describe scaling relationships between leaf flammability and leaf traits and determine whether the scaling relationships differ between native and exotic species.

## Materials and Methods

### Study Species

We assessed a total of 79 plant species (52 native and 27 exotic species) from 35 plant families of dry sclerophyll forest in the greater Sydney region of eastern New South Wales (NSW), Australia. Dry sclerophyll forest is associated with low-fertility soils, low rainfall, sandstone geology, and is one of the most dominant vegetation assemblages in eastern NSW, accounting for nearly one quarter of mapped vegetation in the area [31]. Dry sclerophyll forest is significant in NSW because the south-eastern corner of Australia experiences severe fires due to hot, dry and windy weather conditions [7].

A target list of co-occurring native and exotic plant species found in the abundant mid-storey canopy layer was initially compiled for the study using species inventories detailing vegetation of Sydney’s dry sclerophyll forest communities growing on Hawkesbury Sandstone [32]–[34]. Taxonomic nomenclature followed [35]. We conducted surveys during March and April of 2010 at six sites of dry sclerophyll forest to locate native and exotic plant species on the target list. Sites were selected that were homogeneous with respect to soil type, vegetation structure, aspect, fire history, species composition and surrounding land use (site details in [36]). All sites were located in the Sydney Basin IBRA Bioregion and experience typical Mediterranean climatic conditions.

### Ethics Statement

No specific permissions for site access or plant collection were required for field work at the study sites because they were either on land owned by the University of Technology Sydney or involved the collection of exotic plant species (which does not require a permit in the study region). The field studies did not involve endangered or protected species.

### Leaf Collection and Measurements

Leaves were collected from five healthy replicate plants of each species. Here, the term ‘leaf’ also refers to plant extremities with a photosynthetic role in plants without ‘true leaves’ (e.g. cladodes, phyllodes and branchlets). In the field, leaves were placed inside labelled plastic zip-locked bags that were sealed after all the air had been removed. Bags were then placed between layers of moist newspaper inside a portable insulated polystyrene cooler box which also contained two frozen ice bricks. The cooler was returned to the laboratory within two hours of samples being collected and stored in a cool room kept below 4°C. Measurements of leaf traits were taken within 24 hours of collection and ignition tests of fresh leaves were performed within 48 hours, the time limit within which fresh leaves maintain their characteristics if properly stored [37], [38].

Leaf flammability was measured as time to ignition (ignitability) which refers to the time it takes for a plant structure to ignite after being exposed to an ignition source [19]. Our study focused on single-leaf flammability; while this is only one of several important components contributing to fire behaviour in fire-prone systems, it is a critical component given that leaves are frequently the first structures to ignite during bushfire [18] and will likely dictate much of the behaviour of fire spread through vegetation. Time to ignition of five replicate fresh and five replicate dry leaves (leaves dried at 75°C for 48 hours) was measured using a stop-watch and the radiant heat from a muffle furnace. The method of drying leaves for dry-leaf flammability measurements provided a simple approximation of fallen leaves that have completely dried out in the leaf litter under field conditions. Although leaves in the leaf litter will undoubtedly vary in dryness and nutrient content, the method not only provided an important contrast to fresh-leaf flammability to allow investigation of the importance of leaf water content, it provided a way of comparing dry-leaf flammability among species that ensured consistency in complete water loss. Our dry-leaf flammability measurements have relevance to leaf litter given climatic variability inherent to Mediterranean ecosystems. In very dry years, water content in leaves in the leaf litter could potentially be substantially reduced or even completely removed quite quickly. Thus, our native-exotic comparisons of dry-leaf flammability are most relevant to leaf litter under dry climatic conditions. For clarity in the description and interpretation of leaf flammability patterns, it is important to note that because leaf flammability was measured as time to ignition, a species with leaves with fast ignition times (i.e. low values of ignition time) corresponded to high flammability. In contrast, a species with leaves that took a long time to ignite (i.e. high values of ignition time) had low flammability.

The method used to measure leaf flammability was an adaption of techniques developed by [19] and [23]. Pilot tests were performed to determine what temperature the muffle furnace should be heated to during the experiment to measure leaf flammability. A range of fresh and dry leaves of native and exotic plant species were tested. The temperature of the furnace was measured with an *n*-type thermocouple attached to a DataTaker 600 data logger and data were recorded using DeLogger software (produced by DataTaker). A ceramic fire brick was used to insulate leaves from coming into contact with the muffle furnace, so that ignition would occur only as a result of radiant heat. Pilot tests showed that few leaves ignited in the open muffle furnace at temperatures below 400°C, or only did so after a very long period of time. Although all tested leaves ignited at 800°C, they tended to ignite almost immediately. It was decided that an open-furnace temperature of 500°C, which occurred when the furnace thermostat was set to 700°C, was the optimum test temperature. Following [19], this temperature was chosen for the experimental ignition tests in order that interspecific differences between ignition delay times of leaves could be observed (Gill AM, pers. comm.).

Before ignition tests, the muffle furnace was heated to 700°C over a period of two hours. The furnace reached the required temperature after the first hour; however, the second hour was required to stabilise the temperature. The furnace was heated with its door shut, but testing occurred with the door open and a radiant heat temperature of 500°C. Tests occurred with an open furnace door so that the point of ignition could be observed and to allow the use of a thermocouple to measure radiant heat temperature. Once the furnace temperature had stabilised to 700°C, the furnace door was opened and the ceramic fire brick was slid into the centre of the furnace and a single leaf was placed on the brick with long-handled forceps. The stop-watch was started at the moment the leaf was place on the brick. The point of ignition, at which time the stop-watch was stopped, was defined as the start of pyrolysis (incandescence) in the leaf [22], a point chosen because not all leaves would produce a flame [19].

Five replicate leaves, one from each of the five individual plants, were measured for fresh-leaf flammability, dry-leaf flammability and each of the six leaf traits for each species. Leaf length, width and thickness were measured using Sontax digital vernier callipers with 0.1 mm resolution. Leaf thickness was measured at the widest part of the leaf, at a point two-thirds the distance from the edge of the leaf to the mid-rib. Leaf area was measured as one-sided projected area [38]. Specific leaf area was calculated as leaf area per unit of dry-leaf weight. Percentage leaf water content was estimated as 100×[(fresh-leaf weight – dry-leaf weight)/fresh-leaf weight].

### Analytical Approach

We first tested for differences between natives and exotics in the eight leaf traits including fresh-leaf flammability, dry-leaf flammability, leaf width, leaf area, leaf length, leaf thickness, specific leaf area and % water content of leaves. We then examined scaling relationships between leaf flammability (both fresh-leaf and dry-leaf flammability) and the other six leaf traits and compared scaling relationships for natives with those of exotics. The replicate measurements of each leaf trait were averaged for each species for the analyses. For all leaf traits except % water content of leaves, the averaged data were log-transformed to meet (general) linear modelling assumptions [39]. The bounded nature (0 to 100%) of the data for % water content of leaves indicated the need for logit-transformation of the data [40]. Trait data for the species are presented in the Appendix S1. All analyses were performed using the statistical software R v. 2.15.3, R Development Core Team.

### Comparisons of Leaf Traits between Natives and Exotics

We used a two-way analysis of variance (ANOVA) design in a general linear model to relate leaf flammability to both plant status (native or exotic) and leaf condition (fresh or dry). In this model, leaf flammability was treated as a continuous response variable, plant status was a fixed explanatory variable, leaf condition was a fixed explanatory variable and species were treated as replicates. A significant interaction in the model indicated that leaf flammability differed between natives and exotics contingent on leaf condition. A one-way ANOVA design was then used in separate general linear models to compare the other six leaf traits between natives and exotics. Each leaf trait was treated as a continuous response variable, plant status was a fixed explanatory variable and species were treated as replicates. We also employed phylogenetic logistic regressions to relate each of the eight plant traits to plant status (native or exotic) while explicitly considering the underlying evolutionary relationships among the study species. We first constructed a phylogenetic tree representing the evolutionary relationships among the study species using Phylomatic v. 4.1 [41] based on the APG3 derived megatree R20120829 [42]. The nodes of the phylogeny were then dated [43] and attached to the phylogeny using BLADJ [44]. The phylogenetic tree structure was transformed from multichotomous to dichotomous (binary) format using APE [45]. We performed a series of phylogenetic logistic regressions relating each of the eight leaf traits to plant status using APE [46]. In each separate phylogenetic logistic regression model (hereafter referred to as PLRM), plant status was treated as a binary response variable and a leaf trait was treated as a continuous explanatory variable.

### Scaling Relationships between Leaf Flammability and Leaf Traits

We employed standardized major axis (SMA) regressions using SMATR [47] to examine scaling relationships between leaf flammability (both fresh and dry) and each of the other six leaf traits. First, SMA regression slopes were fitted separately for natives and exotics for each bivariate relationship between leaf flammability and a leaf trait. If the SMA regressions were significant for both natives and exotics for a given bivariate relationship, we then tested the slopes of the relationships for homogeneity using a Bartlett-corrected likelihood ratio test. If the slopes were not significantly different, we tested for significant differences in elevation between the slopes and for significant shifts along the common fitted slope using Wald tests.

## Results

### Comparisons of Leaf Traits between Natives and Exotics

**Figure 1:**
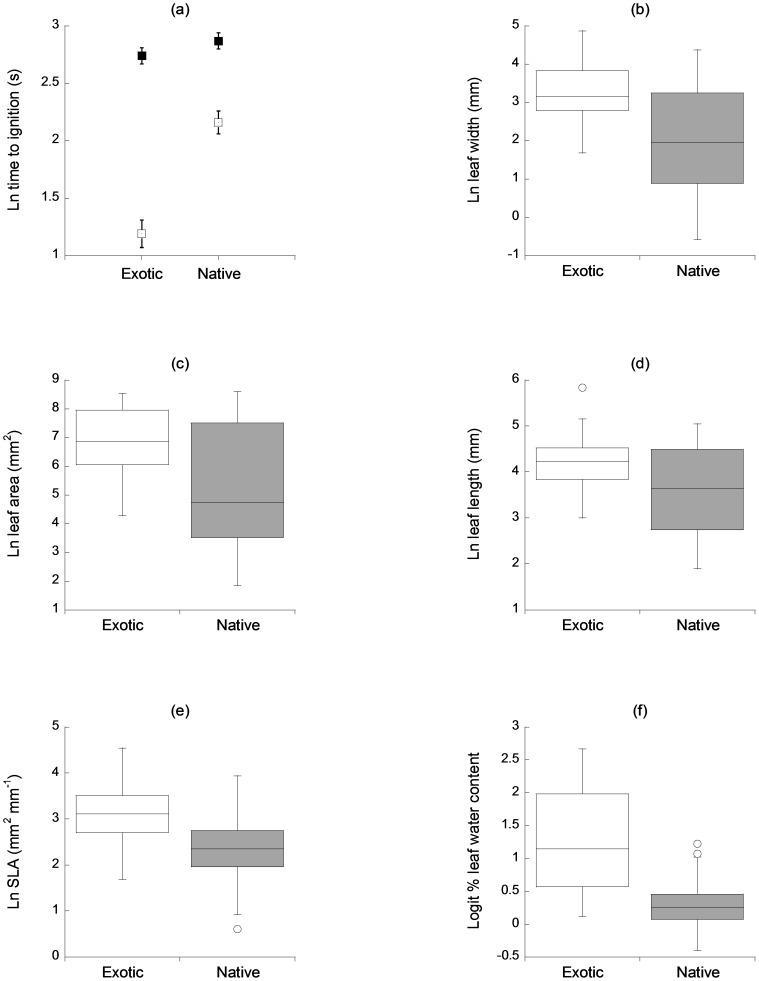
Native-exotic boxplot comparisons of (a) leaf flammability as a function of both plant status (native, exotic) and leaf condition (fresh = closed squares, dry = open squares) and (b) leaf width, (c) leaf area, (d) leaf length, (e) specific leaf area and (f) % water content of leaves.

There was a significant interaction between plant status and leaf condition (*F*_1,154_ = 17.13, *P*<0.001) in the two-way ANOVA model which showed that exotic leaves had significantly faster ignition times than native leaves but only when the leaves were dry (Fig. 1a). This significant interaction overrode the significant individual effects on leaf flammability of plant status (faster ignition times in exotics, *F*_1,154_ = 30.46, *P*<0.001) and leaf condition (faster ignition times in dry leaves, *F*_1,154_ = 111.28, *P*<0.001). Consistent with the pattern represented by the significant interaction, phylogenetic logistic regressions showed that while fresh-leaf flammability did not differ significantly between natives and exotics (*F*_1,77_ = 3.23, *P* = 0.08), dry-leaf flammability was significantly different between natives and exotics (*F*_1,77_ = 27.22, *P*<0.001) with faster ignition times in dry exotic leaves. Compared to native leaves, the leaves of exotics were significantly wider (*F*_1,77_ = 20.62, *P*<0.001, Fig. 1b; PLRM *F*_1,77_ = 17.07, *P*<0.001), broader in area (*F*_1,77_ = 18.58, *P*<0.001, Fig. 1c; PLRM *F*_1,77_ = 17.51, *P*<0.001), longer (*F*_1,77_ = 7.40, *P*<0.01, Fig. 1d; PLRM *F*_1,77_ = 5.58, *P*<0.05), with significantly higher specific leaf area (*F*_1,77_ = 33.55, *P*<0.001, Fig. 1e; PLRM *F*_1,77_ = 47.74, *P*<0.001) and significantly higher % water content (*F*_1,77_ = 49.31, *P*<0.001, Fig. 1f; PLRM *F*_1,77_ = 44.45, *P*<0.001). Leaf thickness did not differ significantly between natives and exotics (*F*_1,77_ = 0.77, *P* = 0.38; PLRM *F*_1,77_ = 3.54, *P* = 0.06).

### Scaling Relationships between Leaf Flammability and Leaf Traits

**Figure 2:**
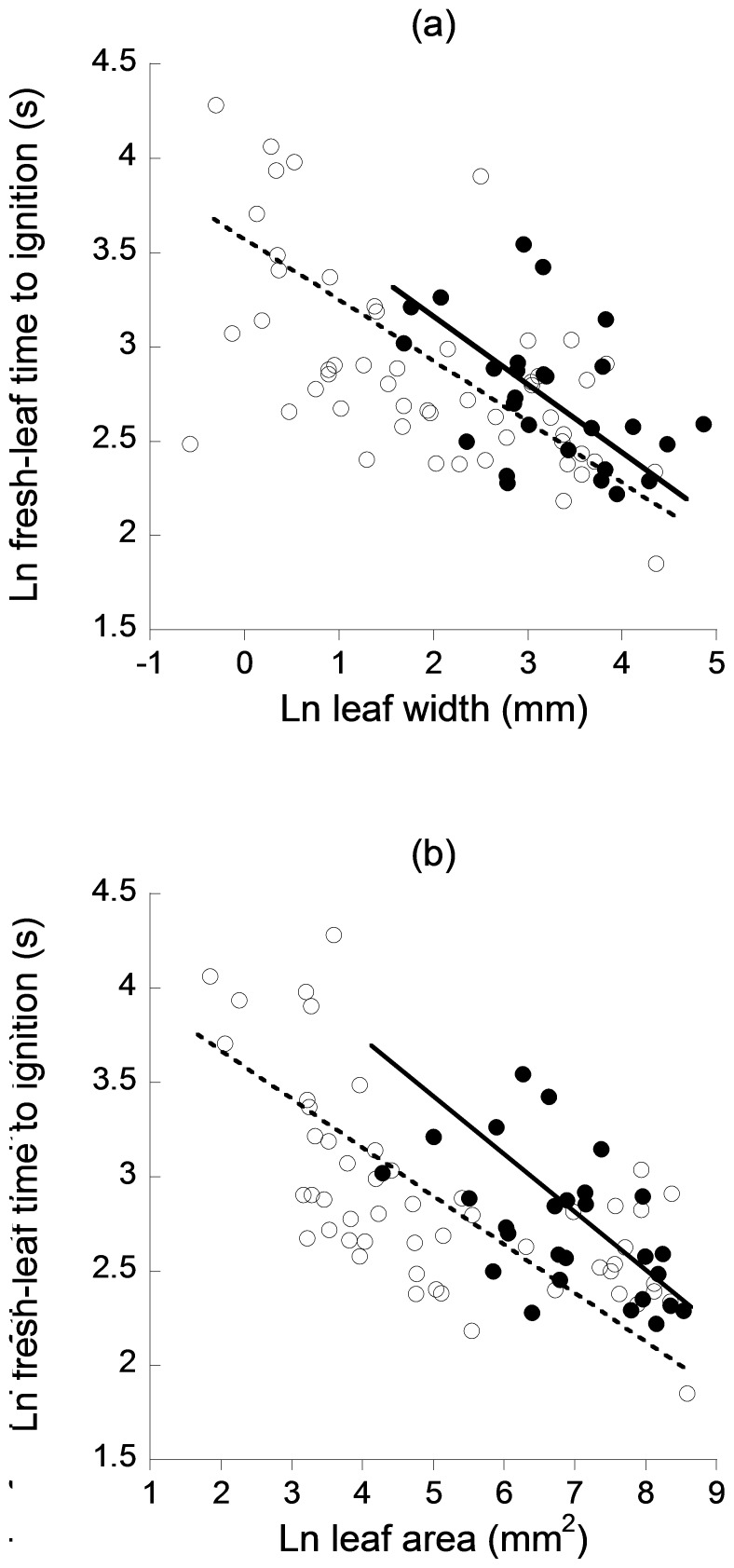
Scaling relationships between fresh-leaf flammability and (a) leaf width and (b) leaf area. Native species are represented by open circles and dashed lines and exotic species by closed circles and solid lines.

Fresh-leaf flammability was significantly correlated with leaf width (natives *r^2^* = 0.36, *P*<0.001, slope = −0.39; exotics *r^2^* = 0.20, *P*<0.05, slope = −0.46) and leaf area (natives *r^2^* = 0.41, *P*<0.001, slope = −0.26; exotics *r^2^* = 0.29, *P*<0.01, slope = −0.34) such that significantly faster ignition times were found in wider (Fig. 2a) and broader (Fig. 2b) leaves. Slopes were homogeneous between natives and exotics for both width (*P* = 0.38) and area (*P* = 0.22), with significant shifts along the slope (width *P*<0.001; area *P*<0.001) and in elevation (width *P*<0.001; area *P*<0.001). Exotics were shifted towards the larger and more flammable end of the spectrum compared with natives; exotics were also slower to ignite than natives for a given leaf width or area. Fresh-leaf flammability was significantly correlated with leaf length in natives (*r^2^* = 0.23, *P*<0.001, slope = −0.55) but not in exotics (*r^2^* = 0.13, *P* = 0.07) and SLA in natives (*r^2^* = 0.17, *P*<0.01, slope = −0.88) but not in exotics (*r^2^* = 0.01, *P* = 0.58). Leaf thickness (natives *r^2^* = 0.03, *P* = 0.25; exotics *r^2^* = 0.13, *P* = 0.07) and % water content (natives *r^2^* = 0.01, *P* = 0.49; exotics *r^2^* = 0.10, *P* = 0.11) were not significantly correlated with fresh-leaf flammability.

**Figure 3:**
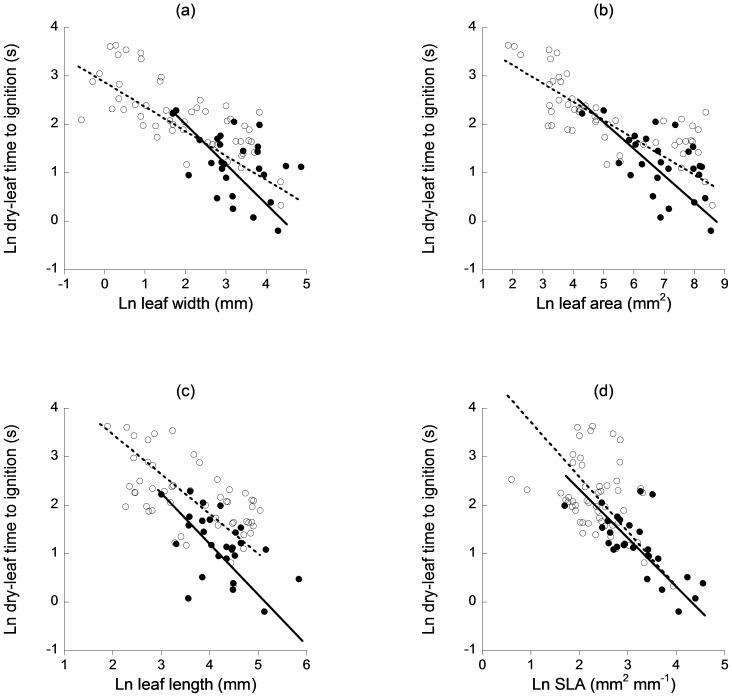
Scaling relationships between fresh-leaf flammability and (a) leaf width, (b) leaf area, (c) leaf length and (d) specific leaf area. Native species are represented by open circles and dashed lines and exotic species by closed triangles and solid lines.

Dry-leaf flammability was significantly correlated with leaf width (natives *r^2^* = 0.54, *P*<0.001, slope = −0.54; exotics *r^2^* = 0.18, *P*<0.05, slope = −0.81), leaf area (natives *r^2^* = 0.57, *P*<0.001, slope = −0.37; exotics *r^2^* = 0.31, *P*<0.01, slope = −0.59), leaf length (natives *r^2^* = 0.34, *P*<0.001, slope = −0.77; exotics *r^2^* = 0.25, *P*<0.01, slope = −1.05) and SLA (natives *r^2^* = 0.13, *P*<0.01, slope = −1.25; exotics *r^2^* = 0.50, *P*<0.001, slope = −0.99) such that significantly faster ignition times were found in wider (Fig. 3a), broader (Fig. 3b) and longer (Fig. 3c) leaves with higher ratios of area to dry weight (Fig. 3d). Slopes were homogeneous between natives and exotics for width (*P* = 0.05), length (*P* = 0.14) and SLA (*P* = 0.23), but not for area (*P* = 0.02), with significant shifts along the slope for width (*P*<0.001), length (*P*<0.001) and SLA (*P*<0.001); exotics were shifted towards the larger, higher-SLA and more flammable end of the spectrum compared with natives. There was a significant shift in elevation for length (*P*<0.01), but not for width (*P* = 0.18) or SLA (*P* = 0.84), with dry exotic leaves faster to ignite than dry native leaves for a given leaf length. The % water content of leaves was significantly correlated with dry-leaf flammability in exotics (*r^2^* = 0.51, *P*<0.001, slope = −0.84), but not in natives (*r^2^* = 0.03, *P* = 0.21), such that exotic leaves with higher water content prior to drying showed faster ignition times when dry. Leaf thickness was not significantly correlated with dry-leaf flammability (natives *r^2^* = 0.01, *P* = 0.46; exotics *r^2^* = 0.04, *P* = 0.34).

## Discussion

We found important differences in leaf flammability between native and exotic plant species inhabiting dry sclerophyll forest. When fresh, leaves of natives and exotics did not differ in mean time to ignition. When dry, however, mean time to ignition was significantly faster for exotics than for natives. Since we found that mean % water content of fresh leaves was significantly higher in exotics than in natives, our results indicate that higher % water content of leaves provides an important buffering effect in exotics, leading to equivalent mean time to ignition in fresh exotic and native leaves. The higher % water content in fresh exotic leaves lengthens their time to ignition because enough heat needs to be absorbed before there is sufficient energy input to vaporise leaf water.

The significantly lower % water content of native leaves compared with exotic leaves, paired with equivalent times to ignition in fresh leaves of natives and exotics, means that there must be other leaf properties that prolong ignition times in native leaves. One possible explanation is that native leaves possess thicker and therefore less permeable cuticles than exotic leaves (noting that overall, mean leaf thickness did not differ between native and exotic leaves). Leaves with less permeable cuticles would lose their water content less easily, take longer to vaporise any leaf oils and subsequently take longer to ignite. There is some evidence that native Australian plant species possess leaves with particularly thick cuticles [48]. Future work comparing cuticle thickness between native and exotic leaves could shed light on the role of leaf permeability in leaf flammability. Furthermore, if a thick cuticle was combined with small leaf size, this combination of leaf properties would lead to even slower rates of water loss from the leaf when exposed to heat. Below, we discuss the role of leaf size as a potential explanation for faster ignition times in large leaves.

Why might mean time to ignition in dry exotic leaves be faster than in dry native leaves? Our results indicate that comparatively larger leaf size observed in exotics plays an important role. We found strong evidence for a relationship between increased leaf flammability and large leaf size in both natives and exotics when fresh and dry. Furthermore, there were shifts along the common slopes of these relationships with exotics occupying the larger more flammable end of the spectrum. We suggest that the link between large leaf size and short time to ignition is due to large leaves having thicker boundary layers [49] and therefore higher average temperatures than small leaves [50]. This makes it harder for a large leaf to lose heat, and hence the larger and hotter the leaf, the easier it ignites. Furthermore, with larger leaves being at a higher temperature than smaller leaves to start with, the bound oils inside leaves will ignite more quickly; and with more of the oils vaporized to the air, ignition becomes even easier. A question for future exploration is whether the absolute amount of ignitable leaf oils is higher in large leaves, perhaps creating the tendency towards ignition sooner. Interestingly, our findings would also seem to indicate that differences in leaf flammability were probably not related to size issues linked to larger leaf mass. This is because the relationships observed between SLA and dry-leaf flammability demonstrate that per unit dry-leaf weight, leaves with larger area were more flammable.

Our findings suggest the possibility of increased fire risk in areas of native vegetation invaded by exotic plant species where exotics are shedding large quantities of dry leaves into the leaf-litter layer. Because we found that dry leaves of exotic species had significantly faster ignition times than dry leaves of native species, exotic species have an intrinsic fuel property (i.e. higher flammability of leaves), which can enhance the spread of fire, that differs from native species. This is of concern because tracts of native vegetation throughout the world are now experiencing situations where native plant species are being displaced by exotic species. Areas that were once dominated by a coexisting suite of native species now either form a matrix of natives and exotics (e.g. [51]) or are replaced with exotic monocultures (e.g. [52]). Indeed, there is substantial evidence that the dry sclerophyll forest that is the focus of this study has experienced and continues to undergo such exotic plant incursions [53], [54].

What this suggests for bushfire spread based on our findings is that shifts in leaf-litter dynamics brought about by the accumulation of dry exotic leaves in the leaf litter might lead to increased bushfire intensity and frequency [11]–[13], [55]. It is not surprising to expect such shifts in bushfire dynamics as it is well known that variation in species composition among plant assemblages provides an indication of fire regimes [55]–[58]. Since dry exotic leaves are more flammable than native leaves, even small additions of the drier exotic leaves are likely to constitute a risk for increased bushfire spread. Worryingly, there is accumulating evidence that exotic plant species can contribute substantial amounts of leaf-fall to the leaf-litter layer [59].

What is needed now for dry sclerophyll forest areas in which there is a matrix of native and exotic species is a thorough quantification of the relative input of exotic to native leaves into the leaf litter. From a management perspective, such information can potentially be tied to quantification of leaf flammability as in the present study to obtain estimates of increased likelihood of bushfire spread as a result of the incursion of exotic plant species. Furthermore, such an approach would permit a broader ecological understanding of the scaling of plant traits to fire behaviour. This is particularly important in areas with high litter accumulation and low decomposition rates, as the composition and volume of the leaf litter is critically important for fire transmission [60].

## Supporting Information

Appendix S1 **Leaf trait data for the study species of Murray, Hardstaff & Phillips “Are there differences in leaf flammability, leaf traits and flammability-trait relationships between native and exotic plants of dry sclerophyll forest?”.** Status refers to the native (N) or exotic (E) status of the species in Australia, fresh-leaf flammability was measured as time to ignition and SLA refers to specific leaf area.
(DOCX)
