# Supplementary material for: Differences in Leaf Flammability, Leaf Traits and Flammability-Trait Relationships between Native and Exotic Plant Species of Dry Sclerophyll Forest
Source: PLoS One. 2013 Nov 18;8(11):e79205. doi: 10.1371/journal.pone.0079205 (PMC3832464; doi:10.1371/journal.pone.0079205)
Supplement: Appendix S1 — Leaf trait data for the study species of Murray, Hardstaff & Phillips “Are there differences in leaf flammability, leaf traits and flammability-trait relationships between native and exotic plants of dry sclerophyll forest?”. Status refers to the native (N) or exotic (E) status of the species in Australia, fresh-leaf flammability was measured as time to ignition and SLA refers to specific leaf area. (DOCX) [file pone.0079205.s001.docx]

**Appendix** Leaf trait data for the study species of Murray, Hardstaff & Phillips “Are there differences in leaf flammability, leaf traits and flammability-trait relationships between native and exotic plants of dry sclerophyll forest?”. Status refers to the native (N) or exotic (E) status of the species in Australia, fresh-leaf flammability was measured as time to ignition and SLA refers to specific leaf area.

| **Species** | **Status** | **Flammability** | **Length** | **Width** | **Thickness** | **Area** | **SLA** | **Water content** |
| --- | --- | --- | --- | --- | --- | --- | --- | --- |
|  |  | (fresh-leaf, s) | (mm) | (mm) | (mm) | (mm^2^) | (mm^2^ mm^-1^) | (%) |
| *Acacia linifolia* | N | 14.230 | 36.820 | 1.600 | 0.300 | 56.529 | 16.436 | 56.940 |
| *Acacia longifolia* | N | 12.424 | 134.060 | 16.100 | 0.340 | 1561.862 | 12.507 | 58.174 |
| *Acacia suaveolens* | N | 17.914 | 77.340 | 5.040 | 0.340 | 222.838 | 6.480 | 66.171 |
| *Acacia terminalis* | N | 24.237 | 11.400 | 4.067 | 0.300 | 33.831 | 7.558 | 53.994 |
| *Acacia ulicifolia* | N | 51.150 | 11.440 | 1.400 | 0.660 | 9.584 | 7.419 | 58.133 |
| *Actinotus helianthi* | N | 8.875 | 30.200 | 29.475 | 0.250 | 255.566 | 14.171 | 57.081 |
| *Actinotus minor* | N | 15.168 | 12.900 | 10.660 | 0.180 | 34.107 | 16.043 | 59.507 |
| *Ageratina adenophora* | E | 13.164 | 88.760 | 61.180 | 0.240 | 2960.811 | 94.119 | 87.917 |
| *Ageratina riparia* | E | 13.312 | 77.360 | 20.240 | 0.280 | 871.618 | 37.537 | 78.102 |
| *Allocasuarina littoralis* | N | 12.004 | 135.200 | 0.560 | 0.560 | 117.364 | 8.671 | 55.374 |
| *Angophera hispida* | N | 18.332 | 113.620 | 46.220 | 0.280 | 4315.802 | 5.058 | 40.118 |
| *Araujia sericifera* | E | 10.514 | 86.140 | 45.520 | 0.320 | 2858.273 | 30.479 | 75.968 |
| *Banksia ericifolia* | N | 30.178 | 16.760 | 1.440 | 0.540 | 24.953 | 7.786 | 54.518 |
| *Banksia marginata* | N | 14.672 | 50.220 | 5.420 | 0.300 | 170.065 | 6.822 | 51.540 |
| *Banksia oblongifolia* | N | 16.656 | 79.640 | 20.820 | 0.440 | 1074.197 | 5.871 | 46.726 |
| *Banksia serrata* | N | 20.836 | 130.940 | 31.940 | 0.400 | 2802.987 | 6.372 | 52.413 |
| *Banksia spinulosa* | N | 17.368 | 67.920 | 2.440 | 0.300 | 111.528 | 5.949 | 51.907 |
| *Bauera rubioides* | N | 24.888 | 15.280 | 3.960 | 0.200 | 27.924 | 17.040 | 58.791 |
| *Billardiera scandens* | N | 10.814 | 33.600 | 7.600 | 0.200 | 166.418 | 20.458 | 62.701 |
| *Boronia ledifolia* | N | 16.500 | 21.380 | 4.580 | 0.460 | 68.534 | 11.510 | 61.236 |
| *Bossiaea heterophylla* | N | 17.800 | 17.440 | 2.440 | 0.300 | 31.780 | 14.823 | 59.190 |
| *Callicoma serratifolia* | N | 10.208 | 116.620 | 35.560 | 0.340 | 2651.576 | 11.708 | 54.295 |
| *Ceratopetalum gummiferum* | N | 13.882 | 64.960 | 14.360 | 0.260 | 549.415 | 14.669 | 58.586 |
| *Chlorophytum comosum* | E | 10.142 | 342.820 | 16.100 | 0.400 | 4211.668 | 30.022 | 90.963 |
| *Cinnamomum camphora* | E | 9.902 | 92.540 | 43.820 | 0.240 | 2430.445 | 13.911 | 56.017 |
| *Coreopsis lanceolata* | E | 18.470 | 173.920 | 18.120 | 0.440 | 1265.019 | 14.934 | 79.977 |
| *Corymbia gummifera* | N | 11.418 | 155.020 | 35.660 | 0.380 | 3371.974 | 6.821 | 48.338 |
| *Cotoneaster glaucophyllus* | E | 17.186 | 47.540 | 24.440 | 0.280 | 826.596 | 11.688 | 53.377 |
| *Dodonaea triquetra* | N | 12.648 | 109.260 | 29.420 | 0.280 | 1936.749 | 15.698 | 70.186 |
| *Elaeocarpus reticulatus* | N | 10.778 | 110.060 | 30.660 | 0.260 | 2054.889 | 10.378 | 53.623 |
| *Eucalyptus haemastoma* | N | 17.177 | 129.617 | 22.483 | 0.400 | 1941.707 | 5.603 | 48.527 |
| *Gonocarpus teucrioides* | N | 19.838 | 11.760 | 8.620 | 0.280 | 65.984 | 27.212 | 77.230 |
| *Grevillea buxifolia* | N | 13.168 | 15.580 | 5.340 | 0.240 | 52.583 | 10.813 | 53.630 |
| *Grevillea linearifolia* | N | 11.066 | 61.820 | 3.660 | 0.160 | 154.191 | 11.151 | 48.820 |
| *Grevillea sericea* | N | 14.146 | 24.240 | 7.160 | 0.300 | 114.766 | 9.012 | 52.950 |
| *Hakea gibbosa* | N | 23.076 | 81.780 | 1.200 | 1.200 | 65.651 | 2.516 | 55.870 |
| *Hakea sericea* | N | 21.570 | 39.400 | 0.880 | 0.880 | 44.106 | 6.452 | 53.964 |
| *Hakea teretifolia* | N | 32.664 | 63.940 | 1.420 | 1.420 | 52.366 | 1.828 | 59.884 |
| *Hedera helix* | E | 11.994 | 77.360 | 88.360 | 0.260 | 3556.102 | 15.981 | 63.910 |
| *Homalanthus populifolius* | N | 10.337 | 84.800 | 77.500 | 0.200 | 4224.870 | 28.170 | 73.461 |
| *Hypochaeris radicata* | E | 17.370 | 88.220 | 23.860 | 0.360 | 1272.427 | 40.574 | 91.597 |
| *Ilex aquifolium* | E | 23.286 | 67.880 | 46.080 | 0.660 | 1583.690 | 5.436 | 56.388 |
| *Jasminum polyanthum* | E | 17.924 | 27.320 | 13.980 | 0.100 | 246.134 | 18.848 | 65.587 |
| *Lantana camara* | E | 9.208 | 91.740 | 51.700 | 0.360 | 3456.876 | 30.064 | 73.491 |
| *Lasiopetalum ferrugineum* | N | 10.996 | 87.080 | 12.800 | 0.440 | 830.434 | 8.925 | 46.512 |
| *Leptospermum arachnoides* | N | 40.644 | 9.900 | 1.140 | 0.340 | 7.816 | 7.091 | 47.245 |
| *Leptospermum squarrosum* | N | 18.266 | 9.660 | 3.540 | 0.200 | 23.604 | 12.593 | 53.369 |
| *Leptospermum trinervium* | N | 14.494 | 10.440 | 2.780 | 0.140 | 24.953 | 11.240 | 41.289 |
| *Ligustrum lucidum* | E | 18.094 | 103.440 | 44.360 | 0.320 | 2853.108 | 11.806 | 65.559 |
| *Ligustrum sinense* | E | 15.348 | 35.700 | 17.540 | 0.220 | 416.033 | 16.187 | 64.557 |
| *Lomandra obliqua* | N | 16.068 | 25.820 | 2.120 | 0.120 | 46.157 | 13.099 | 54.693 |
| *Lomatia silaifolia* | N | 20.780 | 25.100 | 20.100 | 0.300 | 82.062 | 8.314 | 51.006 |
| *Lupinus angustifolius* | E | 24.860 | 36.260 | 5.840 | 0.240 | 148.160 | 25.971 | 82.157 |
| *Micrantheum ericoides* | N | 58.074 | 6.640 | 1.320 | 0.120 | 6.375 | 9.728 | 63.551 |
| *Ochna serrulata* | E | 9.772 | 54.460 | 16.220 | 0.120 | 598.855 | 17.010 | 52.914 |
| *Olea europaea* | E | 12.160 | 46.640 | 10.500 | 0.240 | 344.182 | 13.281 | 54.992 |
| *Osteospermum ecklonis* | E | 34.662 | 56.380 | 19.200 | 0.580 | 528.211 | 18.500 | 88.344 |
| *Oxalis debilis* | E | 13.064 | 34.920 | 39.540 | 0.200 | 964.862 | 80.592 | 88.042 |
| *Passiflora tarminiana* | E | 13.324 | 87.560 | 129.460 | 0.280 | 3778.610 | 22.447 | 73.234 |
| *Persoonia levis* | N | 16.870 | 122.300 | 37.660 | 0.400 | 2809.961 | 7.913 | 64.005 |
| *Persoonia pinifolia* | N | 72.358 | 44.160 | 0.740 | 0.520 | 36.242 | 6.455 | 58.264 |
| *Phyllanthus gunnii* | N | 10.780 | 16.800 | 9.760 | 0.200 | 115.837 | 26.152 | 62.910 |
| *Pimelea linifolia* | N | 29.036 | 15.340 | 2.480 | 0.160 | 25.718 | 17.144 | 72.758 |
| *Pittosporum undulatum* | N | 10.928 | 127.340 | 40.820 | 0.260 | 3385.744 | 7.940 | 57.544 |
| *Plantago lanceolata* | E | 17.758 | 103.440 | 17.960 | 0.300 | 978.339 | 13.476 | 80.528 |
| *Pultenaea stipularis* | N | 53.486 | 25.320 | 1.700 | 0.260 | 24.557 | 9.342 | 60.814 |
| *Rubus ulmifolius* | E | 11.640 | 48.280 | 30.860 | 0.240 | 886.249 | 25.530 | 59.762 |
| *Senecio madagascariensis* | E | 26.158 | 65.400 | 7.980 | 0.360 | 362.953 | 30.897 | 86.346 |
| *Senna pendula* | E | 14.874 | 35.260 | 17.400 | 0.200 | 425.366 | 20.705 | 69.659 |
| *Smilax glyciphylla* | N | 12.150 | 89.220 | 29.100 | 0.380 | 1821.613 | 16.704 | 56.728 |
| *Sonchus oleraceus* | E | 9.874 | 168.740 | 72.760 | 0.280 | 5078.680 | 57.385 | 88.362 |
| *Stephania japonica* | N | 6.354 | 88.380 | 78.680 | 0.280 | 5349.031 | 51.256 | 74.464 |
| *Styphelia tubiflora* | N | 18.218 | 14.800 | 2.600 | 0.180 | 26.776 | 10.533 | 44.634 |
| *Tradescantia fluminensis* | E | 30.722 | 46.640 | 23.680 | 0.420 | 754.888 | 68.409 | 93.527 |
| *Tristaniopsis laurina* | N | 13.824 | 129.860 | 25.600 | 0.360 | 2242.836 | 7.487 | 51.872 |
| *Vicia sativa* | E | 20.462 | 20.100 | 5.400 | 0.160 | 72.524 | 33.512 | 75.931 |
| *Woollsia pungens* | N | 49.690 | 11.600 | 12.200 | 0.180 | 26.381 | 10.970 | 40.524 |
| *Xanthosia pilosa* | N | 16.392 | 26.640 | 20.960 | 0.200 | 258.768 | 24.570 | 69.350 |
| *Xanthosia tridentata* | N | 14.374 | 16.680 | 6.980 | 0.400 | 45.232 | 18.390 | 69.634 |
